# TERT Immunohistochemistry Is a Poor Predictor of *TERT* Promoter Mutations and Gene Expression in Follicular Thyroid Carcinoma

**DOI:** 10.1007/s12022-018-9551-6

**Published:** 2018-10-10

**Authors:** Johan O. Paulsson, Anton Olander, Felix Haglund, Jan Zedenius, C. Christofer Juhlin

**Affiliations:** 1 Department of Oncology-Pathology, Karolinska Institutet, Karolinska University Hospital, CCK, SE-171 76 Stockholm, Sweden; 2 Department of Pathology and Cytology, Karolinska University Hospital, SE-171 76 Stockholm, Sweden; 3 Department of Molecular Medicine and Surgery, Karolinska Institutet, Karolinska University Hospital, SE-171 76 Stockholm, Sweden; 4 Department of Breast, Endocrine Tumours and Sarcoma, Karolinska University Hospital, SE-171 76 Stockholm, Sweden

Dear Editor,

We recently reported genetic and epigenetic aberrations in the telomerase reverse transcriptase (*TERT*) gene in follicular thyroid tumors, further supporting the notion that expression of *TERT* could constitute a prognostic tool for these lesions [1]. We also showed that screening for *TERT* aberrancies showed a high specificity for distinguishing follicular thyroid carcinoma (FTC) and follicular tumor of uncertain malignant potential (FT-UMP) from follicular thyroid adenoma (FTA). Screening for such molecular changes (*TERT* promoter mutations and/or aberrant methylation, copy number gain, and mRNA expression) is, however, not as well established as immunohistochemistry (IHC) in most clinical pathology laboratories. In addition, a reliable TERT antibody has not yet showed satisfactory results in thyroid cancer, with conflicting results in the interpretation of the staining patterns. Moreover, these previous studies were not correlated to various *TERT* gene aberrancies, thereby preventing an essential correlation to underlying genetics. Our aim with this study was to evaluate if TERT immunoreactivity correlates to *TERT* promoter mutations or mRNA expression in FTCs, to see whether TERT IHC could be used as a surrogate marker for the causal genetic analyses that previously have been shown to correlate to a malignant phenotype in follicular tumors. The cohort comprised of 51 FTC cases (denoted T1-T51) operated at Karolinska University Hospital, Stockholm, Sweden, between 2014 and 2016. Of these, 27 were widely invasive (WIFTCs), 17 were minimally invasive (MIFTC) and seven were encapsulated angio-invasive (EAIFTC) according to the 2017 WHO classification algorithm (Table 1). T1-T28 were denoted as part of the validation cohort with known *TERT* promoter mutational status and *TERT* mRNA expression from our previous study [1], and an additional 23 cases (T29-T51) without available genetic information were retrieved from our pathology registries as an expansion cohort. Informed consent was obtained from all individual participants included in the study, and clinical follow-up was retrieved for all patients. Out of 51 FTCs, 38 cases included corresponding normal thyroid tissue on the same slide which allowed comparison of the immunoreactivity with non-tumorous thyroid tissue. All cases were formalin-fixed paraffin-embedded (FFPE) and cut into 4 μm thick sections and stained using the Envision+ Dual Link System-HRP (DAB+) (DAKO, Carpenteria, CA, USA). The tissue slides were incubated with the monoclonal primary antibody anti-telomerase reverse transcriptase (ab32020, Abcam, Cambridge, UK) for 30 min at a dilution of 1:100. As a positive control, primary FUCH-1 (fibroblast cells infected with *TERT* [2] with approximately 10% expressing the protein) were used and BJ cells from human foreskin (ATCC® CRL-2522™, Manassas, VA, USA) were used as a negative control. The optimal primary antibody concentration for our experiments was determined through serial dilutions using positive and negative controls (data not shown). The tissue slides were counterstained with hematoxylin and subsequently evaluated independently by two experienced pathologists and graded as displaying “negative/no immunoreactivity,” “weak immunoreactivity,” or “strong immunoreactivity.” The cell component that showed immunoreactivity was also taken into consideration and described as “nuclear,” “cytoplasmic,” or “perinuclear.”

**Table 1:** TERT immunoreactivity in follicular thyroid carcinoma

			TERT immunoreactivity			
FTC no.	WHO 2017 subtype	Cohort	Tumor tissue	Corresponding normal thyroid tissue	*TERT* promotor mutation	*TERT* mRNA expression
T1	WIFTC	Validation	Weak perinuclear	N/A	Y	Y
T2	MIFTC	Validation	Weak perinuclear	N/A	Y	Y
T3	WIFTC	Validation	Weak perinuclear	Weak perinuclear	Y	Y
T4	WIFTC	Validation	Weak perinuclear	Negative	Y	Y
T5	WIFTC	Validation	Weak perinuclear	N/A	N	Y
T6	WIFTC	Validation	Weak perinuclear	Weak perinuclear	Y	Y
T7	Oxy WIFTC	Validation	Strong perinuclear	Weak perinuclear	N	Y
T8	MIFTC	Validation	Strong perinuclear	N/A	Y	Y
T9	MIFTC	Validation	Strong perinuclear	N/A	N	Y
T10	MIFTC	Validation	Strong perinuclear	Negative	N	Y
T11	WIFTC	Validation	Negative	Negative	Y	Y
T12	WIFTC	Validation	Negative	Weak perinuclear	Y	Y
T13	WIFTC	Validation	Negative	N/A	Y	Y
T14	MIFTC	Validation	Weak perinuclear	Weak perinuclear	N	N
T15	WIFTC	Validation	Weak perinuclear	Weak perinuclear	N	N
T16	EAIFTC	Validation	Strong perinuclear	Negative	N	N
T17	MIFTC	Validation	Strong perinuclear	Weak perinuclear	N	N
T18	WIFTC	Validation	Strong perinuclear	Weak perinuclear	N	N
T19	MIFTC	Validation	Strong perinuclear	Weak perinuclear	N	N
T20	WIFTC	Validation	Strong perinuclear	N/A	N	N
T21	EAIFTC	Validation	Strong perinuclear	Negative	N	N
T22	Oxy EAIFTC	Validation	Strong perinuclear	Weak perinuclear	N	N
T23	WIFTC	Validation	Negative	Weak perinuclear	N	N
T24	WIFTC	Validation	Negative	Negative	N	N
T25	EAIFTC	Validation	Negative	N/A	N	N
T26	WIFTC	Validation	Negative	Weak perinuclear	N	N
T27	WIFTC	Validation	Negative	Weak perinuclear	N	N
T28	MIFTC	Validation	Negative	Negative	N	N
T29	MIFTC	Expansion	Weak perinuclear	N/A	N/A	N/A
T30	EAIFTC	Expansion	Weak perinuclear	Weak perinuclear	N/A	N/A
T31	WIFTC	Expansion	Weak perinuclear	Negative	N/A	N/A
T32	EAIFTC	Expansion	Weak perinuclear	Negative	N/A	N/A
T33	MIFTC	Expansion	Weak perinuclear	Weak perinuclear	N/A	N/A
T34	WIFTC	Expansion	Weak perinuclear	Weak perinuclear	N/A	N/A
T35	MIFTC	Expansion	Weak perinuclear	Weak perinuclear	N/A	N/A
T36	MIFTC	Expansion	Weak perinuclear	Weak perinuclear	N/A	N/A
T37	Oxy MIFTC	Expansion	Weak perinuclear	Weak perinuclear	N/A	N/A
T38	MIFTC	Expansion	Weak perinuclear	N/A	N/A	N/A
T39	WIFTC	Expansion	Weak perinuclear	N/A	N/A	N/A
T40	MIFTC	Expansion	Strong perinuclear	Negative	N/A	N/A
T41	MIFTC	Expansion	Strong perinuclear	Weak perinuclear	N/A	N/A
T42	WIFTC	Expansion	Strong perinuclear	Negative	N/A	N/A
T43	Oxy WIFTC	Expansion	Strong perinuclear	Negative	N/A	N/A
T44	Oxy WIFTC	Expansion	Strong nuclear /cytoplasmic	N/A	N/A	N/A
T45	WIFTC	Expansion	Strong perinuclear	Negative	N/A	N/A
T46	Oxy EAIFTC	Expansion	Strong perinuclear	Weak perinuclear	N/A	N/A
T47	WIFTC	Expansion	Strong perinuclear	Negative	N/A	N/A
T48	WIFTC	Expansion	Strong perinuclear	Weak perinuclear	N/A	N/A
T49	WIFTC	Expansion	Negative	Weak perinuclear	N/A	N/A
T50	MIFTC	Expansion	Negative	Weak perinuclear	N/A	N/A
T51	WIFTC	Expansion	Negative	N/A	N/A	N/A

*EAIFTC* encapsulated angio-ivasive follicular thyroid carcinoma, *MIFTC* minimally invasive follicular thyroid carcinoma, *Oxy* oxyphilic variant, *WIFTC* widely invasive follicular thyroid carcinoma, *Y* yes, *N* no, *N/A* not available

**Fig. 1:**
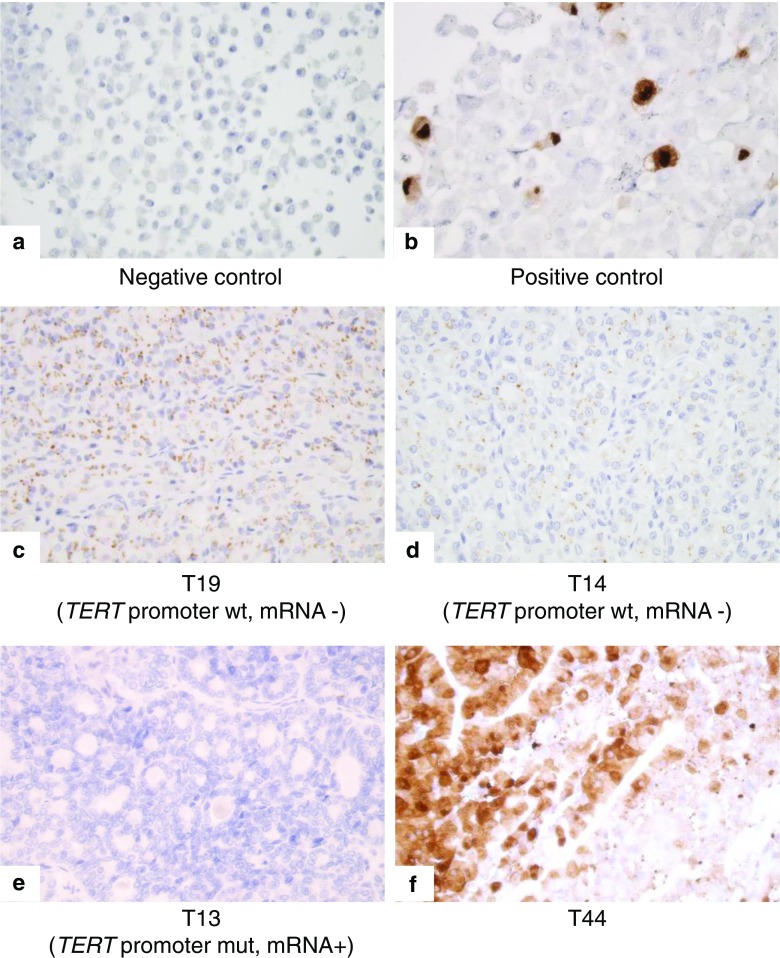
TERT immunoreactivity in controls and follicular thyroid cancer **a** TERT staining of BJ cells shows an expected negative immunoreactivity (× 400 magnification) **b** TERT staining of FUCH1 cells infected with TERT shows strong immunoreactivity in both the nucleus and the cytoplasm of approxiamtely 10% of the cells (× 400 magnification) **c** TERT staining of FTC case T19 (validation cohort case with known absence of *TERT* promoter mutations and mRNA expression) displaying strong perinuclear immunoreactivity (× 400 magnification) **d** TERT staining of FTC case T14 (validation cohort case with known absence of *TERT* promoter mutations and mRNA expression) showing weak perinuclear immunoreactivity (× 400 magnification) **e** TERT staining of FTC case T13 (validation cohort case with an established *TERT* promoter mutation and mRNA expression) with negative immunoreactivity (× 400 magnification) **f** TERT staining of FTC case T44 (from the expansion cohort) with strong immunoreactivity both in the nucleus and cytoplasm (× 400 magnification). This was the only case with positive nuclear staining except for the positive control

The FUCH-1 TERT expressing cells showed strong nuclear and cytoplasmic immunoreactivity whereas the BJ cells showed no immunoreactivity (Fig. 1). Among the FTCs, 20 (39%) showed strong immunoreactivity, 19 (37%) showed weak immunoreactivity and 12 (24%) cases showed negative immunoreactivity (Table 1). A single FTC case (T44) showed nuclear and cytoplasmic staining, whereas all remaining positively stained cases displayed a perinuclear “Golgi-pattern” type of staining of unknown significance (Fig. 1). In the validation cohort, 10 out of 13 FTC cases with known *TERT* mRNA expression displayed either weak or strong TERT immunoreactivity, providing a robust sensitivity for the method. However, among the 15 FTCs in the validation cohort without measurable *TERT* mRNA levels, nine showed either weak or strong TERT immunoreactivity, whereas six were negative—thereby reducing the specificity of the method considerably. Corresponding and adjacent normal thyroid tissues displayed weak perinuclear immunoreactivity in 24 (63%) of cases, and the remaining 14 (37%) showed no immunoreactivity. For statistical purposes, cases with weak immunoreactivity and strong immunoreactivity were collectively regarded as “positive immunoreactivity.” There were no correlations between positive TERT immunoreactivity and *TERT* promoter mutational status or *TERT* mRNA expression (*p* = 1.000 and *p* = 0.435 respectively). Furthermore, when consulting follow-up data of the 51 FTC patients included (data not shown), no increased risk for persistence/recurrences was noted in cases with positive TERT immunoreactivity (*p* = 0.586).

For the first time, we here describe the staining patterns of TERT immunohistochemistry in FTCs, in addition to attempting to correlate the immunoreactivity to established genetic parameters with a known coupling to worse prognosis. All FTCs with positive immunoreactivity except a single case showed an exclusive perinuclear staining which was unexpected when considering the nuclear staining of the positive control in addition to the conventional nuclear role of telomerase. However, recent studies have indeed identified predominant cytoplasmic TERT-staining patterns in other tumor types, for example in hepatocellular carcinoma where cases with cytoplasmic staining surprisingly showed a better overall prognosis [3]. TERT IHC have also been studied in papillary thyroid carcinoma where the protein is hypothesized to shift between subcellular localizations in response to oxidative stress and irradiation [4]. In that study, a “polar” localization was observed in some cases which is in line with our observed perinuclear staining. Moreover, the finding of weak perinuclear staining in the majority of adjacent normal thyroid tissues is puzzling, and might indicate that low cytoplasmic levels of TERT could imply an unknown “house-keeping” function apart from the known roles of TERT in cancer.

The fact that the immunoreactivity in our cohort did not show any correlation to *TERT* mRNA expression in the validation cohort indicates that TERT IHC may not be an ideal surrogate marker for prognostic purposes. While our previous findings indicate that the increase in *TERT* mRNA expression is mainly explained by promoter mutations and aberrant methylation, as well as copy number variations of the *TERT* gene—this was not mirrored by TERT immunoreactivity and indicates that the TERT protein expression displays a much more complex regulation [1]. Several studies have recently been assessing the regulation of TERT, in which further evidence of microRNA regulating mechanisms have been discovered [5]. The lack of concordance between *TERT* mRNA levels and TERT immunoreactivity might suggest post-translational regulation at work, and, therefore, microRNAs targeting *TERT* should constitute ideal candidates for future research purposes in FTCs. Alternatively, the protein turn-over could be discordant with the overall *TERT* mRNA levels in FTCs, arguing for pulsatile expressional cycles of the protein without correlation to the mRNA pool. A third explanation might be that the *TERT* gene aberrancies and subsequent mRNA expression is subclonal, meaning that the area selected for IHC might not represent the area responsible for *TERT* mRNA production.

In conclusion, TERT immunoreactivity does not seem to be a satisfactory marker for prognostication in FTC. Furthermore, the expression pattern and the lack of correlation to mRNA expression indicate a more complex regulation machinery in FTCs that urges to be further studied.
